# Invasive Haemophilus influenzae infections in Germany: impact of non-type b serotypes in the post-vaccine era

**DOI:** 10.1186/1471-2334-9-45

**Published:** 2009-04-20

**Authors:** Helen Kalies, Anette Siedler, Britta Gröndahl, Veit Grote, Astrid Milde-Busch, Rüdiger von Kries

**Affiliations:** 1 Department Epidemiology, Institute for Social Pediatrics and Adolescent Medicine, Ludwig-Maximilians-University, Munich, Germany; 2 Robert-Koch-Institute, Berlin, Germany; 3 Division Paediatric Infectiology, Johannes-Gutenberg-University, Mainz, Germany

## Abstract

**Background:**

Haemophilus influenzae type b (Hib) vaccination led to a significant decrease in invasive bacterial infections in children. The aim of this study was to assess a potential shift to more non-type b invasive infections in a population with high Hib vaccination coverage and to compare the burden of suffering between children with Hib, capsulated non-b and non-capsulated Hi infections.

**Methods:**

Cases with confirmed invasive Hi infections were ascertained through two independent nationwide active surveillance systems in 1998–2005. Information on possible predisposing conditions and clinical information was available from 2001 onwards.

**Results:**

The total number of reported non-type b Hi cases varied between 10 cases in 1998, 27 in 2000 and 14 in 2005. In each year, non-capsulated serotypes outnumbered capsulated non-type b ones. 192 cases were detected in 2001–2005, more than one half was non-type b and 88% of the non-type b cases were non-capsulated. For cases with Hib/capsulated non-type b infections the most common clinical presentation was meningitis (67% each); 89%/78% had no potential predisposing condition, 75%/72% completely recovered from disease and 6% (each) died. In contrast, meningitis was diagnosed in 34% of the non-capsulated Hi infections, septicaemia in 28% and pneumonia 21%; 62% had no potential predisposing condition, 83% completely recovered and 3% died.

**Conclusion:**

There was no increase in non-type b Hi invasive infections during 8 years of active surveillance in Germany. Invasive disease due to non-type b Hi is not confined to children with risk factors. In patients with capsulated non-type b Hi infections the proportion of meningitis cases is similar to Hib, but double as high as in non-capsulated Hi.

## Background

Prior to the development of effective vaccines, *Haemophilus influenzae *(Hi) was one of the most important organisms causing invasive bacterial infections in children in developed countries [1-3]. The predominant serotype causing invasive disease in children was type b (Hib). In Europe, the annual incidence rate of invasive Hib in children less than five years ranged between 20 and 50 per 100,000, with non-type b serotypes being only of anecdotic interest [4-8].

Due to the introduction of conjugate vaccines against Hib the incidence of Hib infections decreased sharply in most countries worldwide [5,9-11]. In Germany, Hib conjugate vaccines were introduced in July 1990 and were subsequently combined with diphtheria, tetanus and acellular pertussis antigens (DTaP/Hib) and higher-valent vaccines, additionally incorporating inactivated polio virus and hepatitis B (DTaP-IPV-HB/Hib). All licensed Hib vaccines in Germany are well tolerated and effective [12] against invasive Hib infections and Hib vaccine coverage rates are high (e.g. 84% of 24-month-old children born 2002–2003 received three doses [13]).

There have been relatively few published studies on the impact of Hib vaccination on the emergence of non-type b invasive Hi infections in children [14-21]. In 2007, a study conducted between 1996 and 2001 in 12 Canadian paediatric tertiary care centres during the era of universal immunisation against Hib showed that two-third of Hi invasive infections were caused by non-b serotypes and that annual numbers of non-type b invasive infections increased over the years[16]. These non-b serotypes presented with significant morbidity and mortality.

The aim of this population based study in Germany was to assess whether a similar increase in non-type b Hi invasive infections can be confirmed in 1998–2005, and to describe the burden of suffering related to non-type b Hi invasive disease as compared to Hib invasive disease in children less than 10 years.

## Methods

### Case definition

Definition of invasive *Haemophilus influenzae *(Hi) cases required hospitalisation for a systemic infectious disease (e.g. meningitis, pneumonia, epiglottitis, septicaemia, cellulitis, septic arthritis) in paediatric hospitals in Germany with isolation of Hi from a normally sterile body site such as blood or cerebrospinal fluid.

### Data sources

Cases of invasive Hi infections in children younger than 10 years were collected from a hospital-based surveillance system (Hospital-ESPED) and a laboratory-based surveillance system (Laboratory-ESPED) over the period 1998–2005. Both systems are voluntary but require active reporting: the absence of cases has to be reported as well and reminders for missing reports are included on a monthly basis.

In Hospital-ESPED, physicians from all paediatric hospitals in Germany report incident cases of a number of rare conditions on a monthly basis since 1992 to a central study office[22]. Reporting on Hi was included since 1998. These reports provide information on an identification number for Hospital-ESPED, location of the reporting hospital, date of birth, age at disease onset, sex, date of disease onset, bacteriologic serotype, detection material, potential predisposing conditions and outcome as well as vaccination information.

For isolation of respective pathogens paediatric hospitals send specimen of body tissue to microbiological laboratories. From 1998–2005, all laboratories throughout Germany serving the paediatric hospitals were asked to report any detection of Hi in physiologically sterile materials in children to Laboratory-ESPED. Collected data were comparable to Hospital-ESPED: identification number for the sample as given by the laboratory, patient's month and year of birth, 3-digit postal code of patient's residence, sex, date of sample arrival at laboratory, bacteriologic serotype, material, and whether an isolate was sent to the National *H. influenzae *Consulting Laboratory for capsule typing, and bacteriologic serotype if available. From 2001 onwards, possible predisposing conditions and clinical information, were also collected by tracing each patient back to the reporting hospital [23].

Cultural confirmation was performed in the local laboratories participating in the surveillance programme according to their routine procedures. Local laboratories also performed PCR testing for capsule type on recovered isolates when available. Laboratories were encouraged to send their specimens to the National *H. influenzae *Consulting Laboratory at the Department of Paediatric Infectious Diseases, Johannes-Gutenberg-University, Mainz, Germany. Capsular typing was performed by slide agglutination using a commercial kit (*Haemophilus influenzae *Agglutinating Sera (a-f); Murex Biotech Ltd., Dartford, UK). The capsular type was confirmed by molecular typing with the primers and methods recommended by the European *Haemophilus *Reference Unit [24]. First, the capsular gene bexA and the b-specific capsular gene were detected by PCR in order to identify b^-^-mutants. If bexA was present the specific gene for the capsular types a-f was detected by PCR. The biotype was determined as described by Kilian [25]. If slide agglutination and PCR results were discordant, PCR results were considered final. If samples for the case were not sent to the reference laboratory but local typing results were available, they were considered final; if samples for the case were sent to the reference laboratory, the reference laboratory results are considered final. If no typing was performed, the case is considered "untyped".

The linkage between the two sources was done by hand applying defined matching rules and using the following patient characteristics: month and year of birth, sex, region of residence (ie, county and federal state vs. address of the hospital), and date of disease onset.

### Analyses

To describe annual trends in the different Hi serotypes we used all cases detected in the two surveillance systems from January 1998 to December 2005. Trends in cases with Hib, non-type b and untyped Hi infections were analysed using a Poisson model. Differentiation in capsulated and uncapsulated non-type b cases was not performed due to the low number of capsulated cases. Since information on potential predisposing conditions and clinical outcome was only available from January 2001 onwards for both sources, we restricted the description of the clinical characteristics for the different serotypes to those cases. For comparison between children with Hib, capsulated non-b and uncapsulated infections, a global test of difference was performed. If results were significant (p < 0.001), cases with capsulated and uncapsulated non-type b infections were pairwise compared to cases with Hib infections.

The study received the positive vote by the ethics committee of the medical faculty of the Ludwig-Maximilians University, Munich.

## Results

### Characteristics of the surveillance systems

Response rates per year ranged between 95% and 98% for Clinical ESPED and between 94% and 100% for Laboratory ESPED. Clinical ESPED detected between 47% (2005) and 66% (2003) of all reported children with invasive Hi disease, and Laboratory ESPED between 79% (2000) and 98% (2001). Serotyping was performed by the National *H. influenzae *Consulting Laboratory between 64% (2000) and 79% (2005) of all Hi cases per year.

### Annual trends of Hi cases

**Figure 1:**
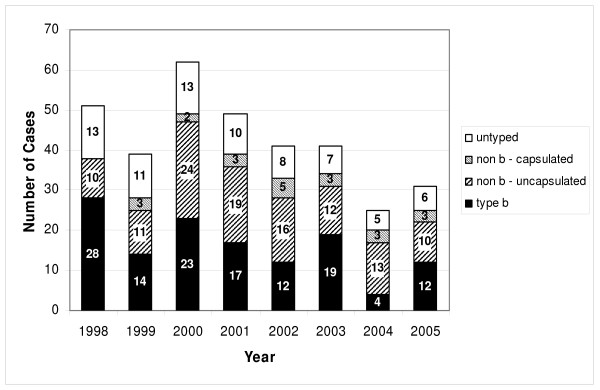
**Annual number of invasive *H. influenzae *cases by serotype detected from 1998 to 2005**.

The total number of reported cases decreased from 51 children in 1998 to 32 in 2005 (Fig. 1). The number of untyped serotypes decreased from 13 cases in 1998 to 6 cases in 2005 (p < 0.001). Overall, type b cases decreased from 28 in 1998 to 12 in 2005 (p < 0.001). There was no trend for non-type b cases (p = 0.771). In most years non-b Hi serotypes outnumbered Hib serotypes, with the exception of 1998, 1999 and 2003. In each year, non-capsulated serotypes outnumbered capsulated non-type b ones (0–5 capsulated non-type b vs. 10–24 non-capsulated per year).

### Clinical characteristics of the different serotypes since 2001

In total, 192 cases of invasive Hi in children were detected from January 2001 to December 2005. Serotyping was performed in 157 of the 192 reported cases (82%). Overall 64 cases were determined to be type b and 93 to be non-type b. Of the 93 non-type b cases, 18 were capsulated, 70 were non-capsulated (a-f negative) and 5 cases were not further specified.

Table 1 summarises the possible predisposing conditions, diagnostic characteristics, and vaccination status of the different Hi serotypes.

**Table 1:** Characteristics of H. influenzaecases by serotype detected from 2001 to 2005 (n = 192).^#^

Number cases/examined cases (% or 10–90^th ^percentile)	Type b N = 64	Non-type b – capsulated N = 18	Non-type b – uncapsulated N = 70
Median age at disease onset (10–90^th ^percentile)	11 (3–40)	31(5–68)	26 (0–73)

Male sex	36/64 (56%)	9/18 (50%)	49/70 (70%)

Diagnosis			
Meningitis	43/64 (67%)	12/18 (67%)	23/68 (34%)***
Pneumonia	5/64 (8%)	3/18 (17%)	14/68 (21%)*
Epiglottitis	6/64 (9%)	0/18 (0%)	0/68 (0%)**
Septic arthritis	1/64 (2%)	1/18 (6%)	0/68 (0%)
Osteomyelitis	1/64 (2%)	0/18 (0%)	0/68 (0%)
Other foci	4/64 (6%)	1/18 (6%)	12/68 (18%)
Septicaemia without focus	4/64 (6%)	1/18 (6%)	19/68 (28%)*

No underlying disease	56/63 (89%)	14/18 (78%)	40/65 (62%)***

Outcome			
recovery	48/64 (75%)	13/18 (72%)	52/63 (83%)
sequelae	12/64 (19%)	4/18 (22%)	9/63 (14%)
Death	4/64 (6%)	1/18 (6%)	2/63 (3%)

Biotype			
I	31/45 (69%)	10/17 (59%)	11/70 (16%)***
II	13/45 (29%)	5/17 (29%)	31/70 (44%)
III	0/45 (0%)	1/17 (6%)	18/70 (26%)
IV	0/45 (0%)	1/17 (6%)	0/70 (0%)
V	0/45 (0%)	0/17 (0%)	8/70 (11%)*
Other	1/45 (2%)	0/17 (0%)	2/70 (3%)*

# if global tests were significant (p < 0.001), capsulated and uncapsulated non-type b cases were pairwise compared to type b cases (* p < 0.05, ** p < 0.01, *** p < 0.001).

#### Serotype b

Of the 64 Hib cases, median age (10^th ^– 90^th ^percentile) at disease onset was 11 (3–40) months. The most common clinical presentation was meningitis, followed by epiglottitis and pneumonia. Potential predisposing conditions were reported in 7 children (11%): 3 with prematurity, 1 child with immunsuppressive therapy, 2 with Down syndrome, and 1 with suspected cerebrospinal fluid (CF) fistula. 48 of the 64 Hib cases completely recovered from acute infection without any obvious sequelae and 4 children died. All children with fatal outcome had meningitis.

For 45 of the 64 Hib cases the specific biotype was measured: 31 had biotype I (68.9%), 13 biotype II (28.9%) and 1 (2.2%) biotype VII. Vaccination status was known for all but one Hib case. 40 were not vaccinated and 23 were vaccinated at least once before disease onset. Of the 23 vaccinated cases, 6 were considered to be vaccine failures of the incomplete primary vaccination schedule, 10 were considered to be vaccine failures of the primary vaccination schedule (2 or 3 doses in the first year of life, depending on the vaccine used) and 7 were considered to be vaccine failures of the full immunisation schedule (primary vaccination plus booster dose or a single dose in the second year of life). Four of the 23 vaccinated cases were reported to have potential predisposing conditions (17%). None of the vaccinated Hib cases died.

#### Capsulated non-b serotypes

Of the 18 capsulated non-type b cases, 15 were type f and 1 each was type a, type c and type e. Median age (10^th ^– 90^th ^percentile) at disease onset was 31 (5–68) months. The most common clinical presentation was meningitis, followed by pneumonia and septic arthritis. For 4 of the 18 children (22%) potential predisposing conditions were reported: one child with congenital rubella syndrome, one with orofaciodigital syndrome, one with cerebrofluid fistula, one with concurrent varicella infection. 13 of the 18 cases completely recovered from acute infection without any obvious sequelae and 1 child died. This 5 month-old girl had meningitis due to a type a infection and no predisposing condition.

For 17 of the 18 capsulated non-b cases the specific biotype was measured: 10 hadbiotype I (58.8%), 5 biotype II (29.4%) 1 biotype III (5.9%) and 1 (5.9%) biotype IV.

#### Non-capsulated serotypes

Of the 70 non-capsulated cases, median age (10^th ^– 90^th ^percentile) at disease onset was 26 (0–73) months. The most common clinical presentation was meningitis, followed by septicaemia without detectable focus and pneumonia. However, meningitis was less common in uncapsulated serotypes compared to Hib serotypes (p < 0.0001). For 25 children (38%) potential predisposing conditions were reported: 12 prematurity, 5 immunodeficiency and/or immunsuppressive therapy, 1 Down syndrome, 3 suspected CF fistula, 1 recurrent pneumonia, 1 gangliosidosis, 1 RSV infection, 1 cardial malformation. 83% of cases completely recovered from acute infection without any obvious sequelae. 2 children died, a preterm (29^th ^week of pregnancy) girl with sepsis and a 22-month old boy with meningitis with a medical history of immunodeficiency. This proportion is lower than in cases with capsulated Hi infections but this difference did not reach statistical significance.

11 of the 70 non-capsulated children with invasive disease had biotype I (15.7%), 31 biotype II (44.3%) 18 biotype III (25.7%), 8 biotype V (11.4%) and 2 biotype VIII (2.9%).

## Discussion

### Annual trend in non-b Hi serotypes

We found no increase of invasive diseases in children caused by non-type b Hi serotypes in a nationwide active surveillance system covering eight years of the post-vaccine-period. This is in contrast to a recently published Canadian study conducted between 1996 and 2001 during the era of universal immunisation against Hib showing that annual numbers of non-type b invasive disease increased over the years [16]. Further literature on the impact of Hib vaccination on the emergence of non-type b isolates as invasive pathogens in children is scarce, but almost all of them showed no increase of non-b serotypes in young children [14,15,17,18,20,21]. An exception is a study done by Ribeiro and colleagues [19], who described an increase of type a Hi invasive infections from 0.02 per 100,000 person years in the year before Hib vaccine introduction to 0.16 per 100,000 person years one year after introduction. Since this 'trend' is based on one single year after vaccine introduction, the interpretation of these data should be done with caution.

### Relevance of non-b Hi serotypes

#### Non-capsulated Hi

In our study, non-b serotypes accounted for the majority of invasive cases. Almost 80% of these were typed as being non-capsulated. Patients infected with non-capsulated serotypes had less meningitis than patients with Hib infections, and the proportion of children with predisposing conditions was higher. Nevertheless, two third had no predisposing conditions, one third suffered from meningitis, 3% died and 22% had serious sequelae. These results are in accordance with the Canadian study [16]. Other studies found a higher case fatality rate [26] and more than 40% were preterm children [15]. In contrast to cases with capsulated Hi infections, biotype II was the most frequently found non-capsulated subtype in our study. This biotype had so far been described as a common subtype that is less virulent and mostly associated with non-invasive disease [27,28]. However, changing virulence factors of non-capsulated serotypes in the post-vaccine era have been discussed [29].

#### Capsulated Hi

The predominant capsulated non-b serotype found in our study was type f (83% of all capsulated non-b serotypes), whereas in the Canadian study type a was the most common capsulated type (60%), followed by type f (26%). The clinical picture of invasive diseases caused by these capsulated non-b serotypes was similar to Hib concerning the severity of the disease (2/3 with meningitis) and outcome (1/5 with sequelae and 6% died). Although there were more predisposing conditions observed and the mean age at infection was higher than for Hib cases, this effect was not statistically significant.

### Strengths and Limitations

Unlike the Canadian study, which was based on 12 paediatric tertiary care centres, our study was based on a population-wide active surveillance system including all levels of care hospitals. Additionally, we used two independent surveillance systems assuring a high completeness of case detection. Capture-recapture estimates for three surveillance systems reporting Hi in Germany and covering years 2001–2005 showed that the two sources, Laboratory- and Hospital-ESPED, detected 83% of all Hi cases [30].

In many surveillance systems, it is very difficult to ensure consistency of case ascertainment over long periods of time because reporting and typing of cases changes over time. In the data presented, response rates to both surveillance systems were high [12,22] and remained relatively constant, but typing of cases changed between 1998 and 2005. A decrease in typing rates could mask a "true" increase of non-type b serotypes. That was not the case: typing rates increased between 1998 and 2005.

We have covered a post-vaccine period of eight years in our study. A potential limitation is the unavailability of detailed data on the situation before the introduction and following the first years after the introduction of Hib vaccines in Germany. Nevertheless, McConnell and co-workers [16] found increasing numbers of non-b serotypes 10 years after the licensure of the first polysaccharide Hib vaccine and 4 years after the implementation of Hib conjugate vaccines in infancy in all territories of Canada.

## Conclusion

There was no increase in non-type b Hi invasive disease during 8 years of active surveillance in Germany. Severe invasive disease due to non-type b Hi is not confined to children with predisposing conditions. Severity of capsulated non-type b Hi infections is similar to Hib whereas the proportion of meningitis in non-capsulated cases was half of that in Hib cases.

## Competing interests

This study is part of a project evaluating vaccine effectiveness against *Haemophilus influenzae *type b which is financially supported by Sanofi Pasteur MSD and GlaxoSmithKline Biologicals (HK, BG, AM-B). AS, VG and RvK declared no conflict of interests.

## Authors' contributions

HK coordinated and designed the study, analysed and interpreted the data, and drafted the manuscript. AS and BG coordinated and designed the study and revised the manuscript critically for important intellectual content. VG and AM-B revised the manuscript critically for important intellectual content. RvK made substantial contributions to conception and design of the study and revised the manuscript critically for important intellectual content. All authors read and approved the final manuscript

## Pre-publication history

The pre-publication history for this paper can be accessed here:

http://www.biomedcentral.com/1471-2334/9/45/prepub

## References

[B1] Tudor-Williams G Frankland J Isaacs D Mayon-White RT MacFarlane JA Rees DG Moxon ER Haemophilus influenzae type b conjugate vaccine trial in Oxford: implications for the United Kingdom Arch Dis Child 1989 64 520 524 2665657 10.1136/adc.64.4.520 PMC1791988

[B2] Murphy JF The introduction of Haemophilus influenzae B (Hib) vaccine [editorial] Ir Med J 1992 85 123 1473940

[B3] Wenger JD Pierce R Deaver K Franklin R Bosley G Pigott N Broome CV Invasive Haemophilus influenzae disease: a population-based evaluation of the role of capsular polysaccharide serotype. Haemophilus Influenzae Study Group J Infect Dis 1992 165 Suppl 1 S34 35 1588172 10.1093/infdis/165-supplement_1-s34

[B4] Reinert P Liwartowski A Dabernat H Guyot C Boucher J Carrere C Epidemiology of Haemophilus influenzae type b disease in France Vaccine 1993 11 Suppl 1 S38 42 10.1016/0264-410X(93)90158-T 8447173

[B5] Hargreaves RM Slack MP Howard AJ Anderson E Ramsay ME Changing patterns of invasive Haemophilus influenzae disease in England and Wales after introduction of the Hib vaccination programme BMJ 1996 312 160 161 8563536 10.1136/bmj.312.7024.160 PMC2349799

[B6] Takala AK Eskola J Palmgren J Ronnberg PR Kela E Rekola P Mäkelä PH Risk factors of invasive Haemophilus influenzae type b disease among children in Finland J Pediatr 1989 115 694 701 10.1016/S0022-3476(89)80644-4 2809900

[B7] Tozzi AE Salmaso S Ciofi degli Atti ML Panei P Anemona A Scuderi G Wassilak SG Incidence of invasive Haemophilus influenzae type b disease in Italian children Eur J Epidemiol 1997 13 73 77 10.1023/A:1007320124502 9062783

[B8] van Alphen L Spanjaard L van der Ende A Schuurman I Dankert J Effect of nationwide vaccination of 3-month-old infants in The Netherlands with conjugate Haemophilus influenzae type b vaccine: high efficacy and lack of herd immunity [see comments] J Pediatr 1997 131 869 873 10.1016/S0022-3476(97)70035-0 9427892

[B9] Garpenholt O Silfverdal SA Hugosson S Fredlund H Bodin L Romanus V Olcén P The impact of Haemophilus influenzae type b vaccination in Sweden Scand J Infect Dis 1996 28 165 169 10.3109/00365549609049069 8792484

[B10] Adams WG Deaver KA Cochi SL Plikaytis BD Zell ER Broome CV Wenger JD Decline of childhood Haemophilus influenzae type b (Hib) disease in the Hib vaccine era [see comments] Jama 1993 269 221 226 10.1001/jama.269.2.221 8417239

[B11] Herceg A The decline of Haemophilus influenzae type b disease in Australia Commun Dis Intell 1997 21 173 176 9222159 10.33321/cdi.1997.21.36

[B12] Kalies H Grote V Siedler A Grondahl B Schmitt HJ von Kries R Effectiveness of hexavalent vaccines against invasive Haemophilus influenzae type b disease: Germany's experience after 5 years of licensure Vaccine 2008 26 2545 2552 10.1016/j.vaccine.2008.03.001 18403069

[B13] Kalies H Grote V Verstraeten T Hessel L Schmitt HJ von Kries R The use of combination vaccines has improved timeliness of vaccination in children Pediatr Infect Dis J 2006 25 507 512 10.1097/01.inf.0000222413.47344.23 16732148

[B14] Progress toward elimination of Haemophilus influenzae type b invasive disease among infants and children – United States, 1998–2000 MMWR Morb Mortal Wkly Rep 2002 51 234 237 11925021

[B15] Heath PT Booy R Azzopardi HJ Slack MP Fogarty J Moloney AC Ramsay ME Moxon ER Non-type b Haemophilus influenzae disease: clinical and epidemiologic characteristics in the Haemophilus influenzae type b vaccine era Pediatr Infect Dis J 2001 20 300 305 10.1097/00006454-200103000-00016 11303834

[B16] McConnell A Tan B Scheifele D Halperin S Vaudry W Law B Embree J Invasive infections caused by haemophilus influenzae serotypes in twelve Canadian IMPACT centers, 1996–2001 Pediatr Infect Dis J 2007 26 1025 1031 10.1097/INF.0b013e31812f4f5b 17984810

[B17] Millar EV O'Brien KL Watt JP Lingappa J Pallipamu R Rosenstein N Hu D Reid R Santosham M Epidemiology of invasive Haemophilus influenzae type A disease among Navajo and White Mountain Apache children, 1988–2003 Clin Infect Dis 2005 40 823 830 10.1086/428047 15736015

[B18] Muhlemann K Balz M Aebi S Schopfer K Molecular characteristics of Haemophilus influenzae causing invasive disease during the period of vaccination in Switzerland: analysis of strains isolated between 1986 and 1993 J Clin Microbiol 1996 34 560 563 8904414 10.1128/jcm.34.3.560-563.1996 PMC228846

[B19] Ribeiro GS Reis JN Cordeiro SM Lima JB Gouveia EL Petersen M Salgado K Silva HR Zanella RC Almeida SC Brandileone MC Reis MG Ko AI Prevention of Haemophilus influenzae type b (Hib) meningitis and emergence of serotype replacement with type a strains after introduction of Hib immunization in Brazil J Infect Dis 2003 187 109 116 10.1086/345863 12508153

[B20] Haemophilus influenzae invasive disease among children aged <5 years – California, 1990–1996 MMWR Morb Mortal Wkly Rep 1998 47 737 740 9746431

[B21] Slack MP Azzopardi HJ Hargreaves RM Ramsay ME Enhanced surveillance of invasive Haemophilus influenzae disease in England, 1990 to 1996: impact of conjugate vaccines Pediatr Infect Dis J 1998 17 S204 207 10.1097/00006454-199809001-00026 9781764

[B22] von Kries R Heinrich B Hermann M German paediatric surveillance unit (ESPED) Monatsschrift Kinderheilkunde 2001 149 1191 10.1007/s001120170044

[B23] Kalies H Verstraeten T Grote V Meyer N Siedler A Schmitt HJ von Kries R Four and one-half-year follow-up of the effectiveness of diphtheria-tetanus toxoids-acellular pertussis/Haemophilus influenzae type b and diphtheria-tetanus toxoids-acellular pertussis-inactivated poliovirus/H. influenzae type b combination vaccines in Germany Pediatr Infect Dis J 2004 23 944 950 10.1097/01.inf.0000141743.74443.73 15602195

[B24] Falla TJ Crook DW Brophy LN Maskell D Kroll JS Moxon ER PCR for capsular typing of Haemophilus influenzae J Clin Microbiol 1994 32 2382 2386 7814470 10.1128/jcm.32.10.2382-2386.1994 PMC264070

[B25] Kilian M A taxonomic study of the genus *Haemophilus*, with the proposal of a new species J Gen Microbiol 1976 93 9 62 772168 10.1099/00221287-93-1-9

[B26] Campognone P Singer DB Neonatal sepsis due to nontypable Haemophilus influenzae Am J Dis Child 1986 140 117 121 3484894 10.1001/archpedi.1986.02140160035025

[B27] Harper JJ Tilse MH Biotypes of Haemophilus influenzae that are associated with noninvasive infections J Clin Microbiol 1991 29 2539 2542 1774261 10.1128/jcm.29.11.2539-2542.1991 PMC270369

[B28] Raymond J Armand-Lefevre L Moulin F Dabernat H Commeau A Gendrel D Berche P Nasopharyngeal colonization by Haemophilus influenzae in children living in an orphanage Pediatr Infect Dis J 2001 20 779 784 11734741 10.1097/00006454-200108000-00012

[B29] O'Neill JM St Geme JW 3rd Cutter D Adderson EE Anyanwu J Jacobs RF Schutze GE Invasive disease due to nontypeable Haemophilus influenzae among children in Arkansas J Clin Microbiol 2003 41 3064 3069 12843045 10.1128/JCM.41.7.3064-3069.2003 PMC165342

[B30] Milde-Busch A Kalies H Ruckinger S Siedler A Rosenbauer J von Kries R Surveillance for Rare Infectious Diseases: is one passive data source enough for Haemophilus influenzae? Eur J Public Health 2008 18 371 5 10.1093/eurpub/ckn023 18424469

